# A Dog’s Love

**Published:** 1870-07

**Authors:** 


### A DOG'S LOVE.

Some of the most degrading epithets applied to man, associate him with the animal creation. A filthy glutton is a pig. If he is a poor, spiritless individual he is said to be sheepish; a jackass, if he is a noisy fool; a goose, if he has no sense, no reason in him, arising probably from the asserted fact that if a goose enters a gate, whether one foot or forty high, the head is sure to be bowed or bent downwards.

The peacock is vain. It is not remembered that any degrading comparison has ever been made in reference to the horse; by common consent the word "noble" belongs to him. But a "mean dog" is intended to place the person to whom it is applied at the very lowest round of human degradation; and yet the dog is the most faithful of all the animals on earth, and is probably the most sagacious. "A knowing dog" is a very expressive epithet. The dog has been many a time known to have died on the grave of his master, in irreconcilable grief.

One bitter cold night in January, a family at supper heard the loud bark of a dog of the Bernard breed, but paid no attention to it; after an interval he tried to get in at the door, then at the windows; finally, the master opened the door, when the dog immediately ran, the man following him until they came to the big road, where they found a wagoner with his head caught under the heavy wagon body, with life almost extinct; he was extricated, revived after a while, and was taken care of until he was able to return home.

In a case of alarming illness a man wrote a note, tied it around a tan terrier's neck, saying, "Go, sir, to Elmira's." The little fellow trotted off, reached the place, distant seven miles, over a difficult and winding road; the letter was noticed and promptly attended to, the distance having been travelled between three and five o'clock.

Who shall not say that cruelty to one of these fathful and sagacious animals is one of the meanest and cowardliest of crimes?

